# Sleep duration during the COVID-19 pandemic in Bangladesh: A GIS-based large sample survey study

**DOI:** 10.1038/s41598-023-30023-1

**Published:** 2023-02-27

**Authors:** Firoj Al-Mamun, Nur Hussain, Najmuj Sakib, Ismail Hosen, Istihak Rayhan, Abu Hasnat Abdullah, A. K. M. Israfil Bhuiyan, Md. Abedin Sarker, Sahadat Hossain, Liye Zou, Md. Dilshad Manzar, Chung-Ying Lin, Md. Tajuddin Sikder, Mohammad Muhit, Amir H. Pakpour, David Gozal, Mark D. Griffiths, Mohammed A. Mamun

**Affiliations:** 1 CHINTA Research Bangladesh, Savar, Dhaka, Bangladesh; 2 Department of Public Health and Informatics, Jahangirnagar University, Savar, Dhaka, Bangladesh; 3 Department of Public Health, University of South Asia, Dhaka, Bangladesh; 4 Department of Microbiology, Jashore University of Science and Technology, Jashore, Bangladesh; 5 Department of Economics, Jahangirnagar University, Savar, Dhaka, Bangladesh; 6 Department of Behavioural Science and Health, Institute of Epidemiology and Health Care, University College London (UCL), London, UK; 7 Body-Brain-Mind Laboratory, School of Psychology, Shenzhen University, Shenzhen, China; 8 Department of Nursing, College of Applied Medical Sciences, Majmaah University, Al Majma’ah, Saudi Arabia; 9 Institute of Allied Health Sciences, College of Medicine, National Cheng Kung University, Tainan, Taiwan; 10 Social Determinants of Health Research Center, Research Institute for Prevention of Non-Communicable Diseases, Qazvin University of Medical Sciences, Qazvin, Iran; 11 Department of Child Health and The Child Health Research Institute, The University of Missouri School of Medicine, Columbia, MO USA; 12 International Gaming Research Unit, Psychology Department, Nottingham Trent University, Nottingham, UK; 13 Department of Public Health, Daffodil International University, Dhaka, Bangladesh; 14 Present Address: School of Earth, Environment & Society, McMaster University, Hamilton, Canada; 15 Department of Nursing, School of Health and Welfare, Jönköping University, Jönköping, Sweden

**Keywords:** Diseases, Medical research, Risk factors

## Abstract

Although several studies have been conducted in Bangladesh regarding sleep problems during the COVID-19 pandemic, none have utilized a large nationwide sample or presented their findings based on nationwide geographical distribution. Therefore, the aim of the present study was to explore the total sleep duration, night-time sleep, and daily naptime and their associated factors as well as geographic information system (GIS) distribution. A cross-sectional survey was carried out among 9730 people in April 2020, including questions relating to socio-demographic variables, behavioral and health factors, lockdown, depression, suicidal ideation, night sleep duration, and naptime duration. Descriptive and inferential statistics, both linear and multivariate regression, and spatial distribution were performed using Microsoft Excel, SPSS, Stata, and ArcGIS software. The results indicated that 64.7% reported sleeping 7–9 h a night, while 29.6% slept less than 7 h nightly, and 5.7% slept more than 9 h nightly. 43.7% reported 30–60 min of daily nap duration, whereas 20.9% napped for more than 1 h daily. Significant predictors of total daily sleep duration were being aged 18–25 years, being unemployed, being married, self-isolating 4 days or more, economic hardship, and depression. For nap duration, being aged 18–25 years, retired, a smoker, and a social media user were at relatively higher risk. The GIS distribution showed that regional division areas with high COVID-19 exposure had higher rates of non-normal sleep duration. Sleep duration showed a regional heterogeneity across the regional divisions of the country that exhibited significant associations with a multitude of socioeconomic and health factors.

## Introduction

The novel coronavirus disease 2019 (2019-nCoV or COVID-19) emerged in December 2019 and this unexpected and life threatening event has disrupted individuals’ way of life and confined many to living from home. Individuals still do not know how long the pandemic will last, and it has become an unprecedentedly stressful situation^1,2^. Due to the pandemic, psycho-emotional problems have been observed worldwide. For instance, mental health problems such as depression, anxiety, post-traumatic symptoms, sleep problems, and adjustment disorders have been reported globally among various age groups^3,4^. The pandemic led many governments to implement non-pharmaceutical measures to minimize the spread of the disease, such as spatial distancing, quarantining, and self-isolation. Such measures can exacerbate individuals’ vulnerability to loneliness, which may also increase mental health burdens and associated problems^4^. Other consequences related to the pandemic, such as economic distress, financial hardship, and poverty, can also increase the risk of mental health issues^5,6^. Mental health problems are frequently associated with insomnia and sleep duration, and sleep has a bidirectional relationship with the body's physiological system and psychological well-being^7–11^.

A previous study conducted among adults with Human Immunodeficiency Virus (HIV) from the USA reported that sleep duration was associated with telomere length. This study concluded that a minimum of seven hours of nightly sleep may prevent telomere damage or repair them on a nightly basis^12^. Narcolepsy-like sleep problems were also reported after the H1N1 influenza pandemic^13^. In addition, traumatic life events such as wildfires may also exacerbate sleep problems^14^. Similarly, in the context of the COVID-19 pandemic, changes in sleep duration have been reported in a large number of studies. This is not surprising given the fact that sleep problems will increase in response to natural or manmade stressful events^15^. Sleep problems during the COVID-19 pandemic were observed to be high in recent meta-analyses^16,17^. A global study recruiting participants from 49 countries indicated a significant escalation in sleep problems. During the pandemic, the average sleep duration per night also increased compared to before the pandemic (that is, 7.2 ± 1.6 h in COVID-19, 6.9 ± 1.1 h before COVID-19)^18^. Likewise, a couple of studies reported worsening sleep quality during the lockdown period^18–20^. Additionally, shifting in bedtime and waking time, reduction in the number of hours of sleep at night-time, and increasing daytime naps have become more prevalent than before the lockdown period^19^. A study among fitness coaches found that home confinement negatively affected objective measurements of sleep parameters such as sleep latency and total sleep duration, as well as deep and light sleep times^21^. In addition, a decrease from 61 to 48% in good sleep was estimated during home confinement^22^, and such sleep alterations were associated with mental health problems^23^.

The home confinement situation can lead an individual to use social media more frequently and intensify the risk of overusing electronic devices^24^. It has been found that sleep disturbances have been more common among excessive smartphone users^25^. Moreover, individuals' poor health conditions can also increase the risk of sleep disturbance^26^. Several studies during the pandemic have examined insomnia and sleep disturbances in Bangladesh, but none of them investigated sleep duration and its associated factors utilizing large samples^27–29^. Furthermore, geographical differences in terms of sleep duration were absent in these studies. It is worth mentioning that before the COVID-19 pandemic, total sleep time among Bangladeshi people had been investigated by Yunus et al.^30^. It was reported that 87.4% of those aged 18–64 years reported that their sleep time was 7–9 h nightly, 8.9% less than 7 h, and 3.7% more than 9 h. Therefore, the present study investigated the total sleep duration, night-time sleep, and daily naptime and its associated factors among Bangladeshi residents during the COVID-19 pandemic. The study also investigated division-wise sleep duration heterogeneity based on geographic information system (GIS) distribution.

## Methods

### Study procedure, participants, and ethics

This cross-sectional study was conducted during the first phase of the COVID-19-related lockdown in April, 2020 among 9730 people, and data were collected nationwide via online participation. Participants were recruited if they were currently residing in Bangladesh and were aged 18–64 years. Approximately 250 research assistants from all 64 districts of the country circulated online survey forms to ensure a country-wide sample of participants. This study used a non-probability convenience sampling approach. The study followed the standards of ethical practice in the Helsinki Declaration, 1975. In addition, formal IRB approval was obtained before study implementation from the Institute of Allergy and Clinical Immunology of Bangladesh [IRBIACIB/CEC/03202005], and the Jahangirnagar University ethics boards [BBEC, JU/M 2O20/COVlD-l9/(9)2]. An online consent form was provided to the participants. They were informed about the ethical issues, study aims, their non-beneficiary involvement, and right of withdrawal.

### Measures

#### Sociodemographic information

Socio-demographic information such as participants' age, gender, occupation (i.e., employed, student, retired, housewife, unemployed, others), residence (i.e., village, Upazila town, district level town, divisional city), marital status (single, married, divorced/widowed, other) was collected. Additionally, if someone came home from a COVID-19 affected country, it was also recorded.

#### Behavior and health-related information

Behavior-related and health-related information was collected based on binary response (yes/no) concerning cigarette smoking, alcohol consumption, presence of chronic disease, and frequency of social media use. In addition, the participants provided information on self-rated current health conditions (i.e., good, fair, poor).

#### Lockdown-related financial information

The impact of lockdown on finances was assessed. For instance, participants were asked if they (1) felt isolated, (2) had face-to-face contact with another person for at least 15 min, (3) been outside for 15 min daily, (4) had enough food if the lockdown lasted for more than 1 month, (5) would face economic hardship, and (6) would panic about probable economic recession. The first three items were categorized as not a single day, less than four days, and four days or more. The rest of the items were categorized as agree, disagree, and undecided.

#### Depressive symptoms

Participants’ depression was assessed using the nine-item Bangla version of the Patient Health Questionnaire (PHQ-9)^31^, where items were recorded on a four-point scale (0 = not at all, to 3 = nearly every day) with scores ranging from 0 to 27. A score of 10 was used as the cutoff score to indicate depression^32,33^. Cronbach's alpha was 0.83 in the present study.

#### Suicidal ideation

Suicidal ideation was assessed by asking the question (answered yes/no) *“Do you think about committing suicide, and are these thoughts persistent and related to COVID-19 issues?”* (consistent with previous studies^34,35^.

#### Night sleep duration

Nightly sleep duration was assessed based on three categories. These were (1) recommended sleep duration = 7–9 h^36^, (2) longer sleep duration = more than 9 h, and (3) shorter sleep duration = less than 7 h. Longer and shorter sleep duration was categorized following a previous study conducted in the country^30^.

#### Naptime duration

Information related to daily nap time was also assessed. Participants' naptime duration was categorized as no naptime, less than 30 min, 30–60 min, and more than an hour.

### Statistical analysis

Data were analyzed using commercially available statistical software (SPSS 25.0), Stata 16 for descriptive statistics and logistic models, and ArcGIS 10.7 software for spatial analysis of COVID-19 cases and sleep patterns. First-order analyses such as independent *t*-tests, one-way analysis of variance (ANOVA), and chi-square tests were performed to examine the relationship between study variables and dependent variables (total daily sleep time in 24 h, naptime, and nightly sleep duration) using Bonferroni correction (p = 0.002). Here, total daily sleep time was generated, adding both naptime and nightly sleep duration. Later, the significant variables were included for regression analysis. Normality and multicollinearity of the data were also checked for conducting regression analysis. Statistical significance at *p* < 0.05 with a 95% confidence interval was applied in the present study. The GIS mapping was performed using the ArcGIS 10.7 software which explored spatial distribution of sleep patterns. The collected geographic locational data of each respondent/sample was synchronised by district scale and distributed in map in terms of napping, night sleep duration, total sleep duration, night sleep duration, and number of COVID-19 cases.

## Results

### Characteristics of the participants

The demographic characteristics of the participants are presented in Table 1. Of the 9730 participants, 56.0% were male (n = 5452), 43.80% were female (n = 4263), and 0.2% participants were transgender (n = 15). The median age of the participants was 24 years (age range = 18–64 years). The most represented age range was 18–25 years (63.40%). Most of the participants (n = 5674) were students (58.3%), and employees constituted the second-largest group (26.1%). For residential status, 40.50% lived in a divisional city (n = 3941), 23.20% lived in a district-level town (n = 2253), 22.90% lived in a village (n = 2243), and the remainder lived in an Upazila [sub-district] setting (Table 1).

**Table 1:** Association between the study variables and total daily sleep duration (in minutes/24 h).

Variable	Total n (%)	TDSD	Statistics	
		Mean ± SD	*t*/F value	*p*-value
Socio-demographic variables				
Age				
18–25 years	6167 (63.4)	492.46 ± 92.16	61.346	< 0.001
26–35 years	2506 (25.8)	476.34 ± 85.12		
More than 35 years	1057 (10.9)	464.39 ± 82.29		
Gender				
Male	5452 (56)	481.77 ± 88.23	10.255	< 0.001
Female	4263 (43.8)	489.82 ± 91.80		
Transgender	15 (0.2)	459.33 ± 97.94		
Occupation				
Employed	2547 (26.2)	470.21 ± 84.55	28.123	< 0.001
Student	5674 (58.3)	492.90 ± 91.89		
Retired	52 (0.5)	481.53 ± 95.81		
Housewife	689 (7.1)	472.39 ± 82.41		
Unemployed	348 (3.6)	501.53 ± 91.59		
Others	420 (4.3)	481.48 ± 89.76		
Residence				
Village	2227 (22.9)	488.69 ± 85.97	4.288	0.005
Upazila town	1309 (13.5)	490.54 ± 93.10		
District-level town	2253 (23.2)	484.32 ± 90.39		
Divisional city	3941 (40.5)	482.11 ± 90.62		
Marital status				
Single	6861 (70.5)	488.83 ± 91.75	12.742	< 0.001
Married	2753 (28.3)	477.08 ± 84.32		
Divorced/widowed	96 (1.0)	471.07 ± 88.31		
Other	20 (0.2)	456.0 ± 121.02		
Someone coming home from COVID-19 affected country				
Yes	245 (2.5)	497.78 ± 95.02	2.208	0.027
No	9485 (97.5)	484.94 ± 89.76		
Behavior-related variables				
Smoker				
Yes	1449 (14.9)	490.81 ± 92.02	2.548	0.011
No	8281 (85.1)	484.29 ± 89.51		
Alcohol consumer				
Yes	258 (2.7)	490.79 ± 93.03	1.001	0.317
No	9472 (97.3)	485.11 ± 89.82		
Social media user				
Yes	8654 (92)	486.56 ± 90.45	5.264	< 0.001
No	776 (8.0)	470.27 ± 82.0		
Lockdown-related questions				
Self-isolation				
Not a single day	738 (7.6)	476.32 ± 86.20	8.183	< 0.001
Less than 4 days	474 (4.9)	474.31 ± 82.88		
4 days or more	8518 (87.5)	486.65 ± 90.52		
Had face-to-face contact with another person for 15 min or more				
Not a single day	4904 (50.4)	488.54 ± 91.01	7.468	0.001
Less than 4 days	3352 (34.5)	480.79 ± 88.16		
4 days or more	1474 (15.1)	484.52 ± 89.77		
Been outside for 15 min				
Not a single day	4747 (48.8)	488.13 ± 92.05	5.660	0.003
Less than 4 days	3135 (32.2)	483.88 ± 86.35		
4 days or more	1848 (19.0)	480.24 ± 90.05		
Have enough food supply				
Agree	1934 (19.9)	488.01 ± 88.39	3.080	0.046
Disagree	3735 (38.4)	482.48 ± 89.38		
Undecided	4061 (41.7)	486.26 ± 91.06		
May face economic hardship				
Agree	4197 (43.1)	485.66 ± 90.77	8.787	< 0.001
Disagree	1339 (13.8)	474.98 ± 87.27		
Undecided	2126 (21.8)	487.24 ± 90.09		
Panic of probable economic recession				
Agree	8569 (88.1)	485.80 ± 89.32	1.694	0.184
Disagree	596 (6.1)	483.62 ± 93.26		
Undecided	565 (5.8)	478.84 ± 95.02		
Health-related variables				
Depressive symptoms				
Non-depressed	6478 (66.6)	482.68 ± 94.85	4.002	< 0.001
Depressed	3252 (33.4)	490.41 ± 94.85		
Self-rated health				
Good	6729 (69.2)	483.27 ± 88.41	5.732	0.003
Fair	2697 (27.7)	489.25 ± 92.61		
Poor	304 (3.1)	493.97 ± 97.0		
Chronic diseases				
No	7398 (76.0)	485.58 ± 89.20	0.629	0.529
Yes	2332 (24.0)	484.24 ± 92.13		
Suicidal ideation				
Yes	488 (5.0)	500.23 ± 103.18	3.309	0.001
No	9242 (95.0)	484.47 ± 89.09		

TDSD, total daily sleep duration; SD, standard deviation.

### Prevalence of sleep duration

Just over two-fifths of participants (43.7%) reported having a nap duration of 30–60 min daily (n = 4256), whereas 20.9% had a nap duration of more than an hour daily (n = 2034), 3.0% had a nap duration of fewer than 30 min (n = 289), and 2.40% had no daily nap at all (n = 3151). In addition, most of the participants (n = 6296, 64.7%) were sleeping 7–9 h nightly, whereas 29.6% slept less than 7 h nightly (n = 2882) and 5.7% slept more than 9 h nightly (n = 552).

### Association between study variables and total daily sleep duration

The total daily sleep time of the participants was presented as mean score and standard deviation in Table 1. Participants aged 18–25 years showed significantly higher mean scores than any other age group (F = 64.346, *p* < 0.001). Females, those unemployed, and those who were single had significantly higher mean scores in terms of total daily sleep time (*p* < 0.001). Social media users were more prone to having an increase in total daily sleep than non-users (*t* = 5.264, *p* < 0.001). In addition, participants who were socially isolated for four days or more, had no face-to-face contact at all with anyone for 15 min or more, and those facing economic hardship had an increased total daily mean sleep time (*p* < 0.001). Furthermore, those having depressive symptoms (*t* = 4.002, *p* < 0.001), and those experiencing suicidal ideation (*t* = 3.309, *p* < 0.001) had a greater mean score than those who did not in terms of total daily sleep time.

### Association between study variables and daily naptime

Participants' daily naptime association with the study variables is shown in Table 2. The age group had a significant relationship with naptime, with those aged 18–25 years reporting significantly more naptime than other age groups (χ^2^ = 323.736,* p* < 0.001). Gender had a statistically significant association with naptime with males reporting more naptime than females and transgender individuals (χ^2^ = 61.152,* p* < 0.001). Students reported significantly more naptime than others occupations (χ^2^ = 422.597,* p* < 0.001). Naptime duration was also significantly higher among single participants than other relationship status groups (χ^2^ = 223.616,* p* < 0.001). In addition, non-smokers, non-alcohol consumers, and social media users all reported more naptime than their non-user counterparts (*p* < 0.001). Participants not having face-to-face contact with another person for 15 min or more in a single day, not being outside for more than 15 min in a single day, running out of food, and economic recession was also found to have a significant relationship with naptime (*p* < 0.001). Furthermore, participants who were non-depressed (χ^2^ = 15.898, *p* = 0.001), not suffering from a chronic medical condition (χ^2^ = 22.038, *p* < 0.001), and not having suicidal ideation (χ^2^ = 18.350, *p* < 0.001) all had significantly more naptime than their counterparts.

**Table 2:** Associations between the study variables and daily naptime.

Variable	Naptime				Statistics	
	No nap (3151, 2.4%); n (%)	< 30 min (289, 3.0%); n (%)	30–60 min (4256, 43.7%); n (%)	> 60 min (2034, 20.9%); n (%)	*χ*^*2*^ value	*p*-value
Socio-demographic variables						
Age						
18–25 years	1688 (53.6)	167 (57.8)	2748 (64.6)	1564 (76.9)	323.736	< 0.001
26–35 years	978 (31.0)	87 (30.1)	1061 (24.9)	380 (18.7)		
More than 35 years	485 (15.4)	35 (12.1)	447 (10.5)	90 (4.4)		
Gender						
Male	1921 (61.0)	144 (49.8)	2262 (53.1)	1125 (55.3)	61.152	< 0.001
Female	1222 (38.8)	143 (49.5)	1990 (46.8)	908 (44.6)		
Transgender	8 (0.3)	2 (0.7)	4 (0.1)	1 (0)		
Occupation						
Employed	1157 (36.7)	71 (24.6)	984 (23.1)	335 (16.5)	422.597	< 0.001
Student	1490 (47.3)	166 (57.4)	2562 (60.2)	1456 (71.6)		
Retired	12 (0.4)	2 (0.7)	26 (0.6)	12 (0.6)		
Housewife	251 (8.0)	28 (9.7)	349 (8.2)	61 (3.0)		
Unemployed	106 (3.4)	8 (2.8)	144 (3.4)	90 (4.4)		
Other	135 (4.3)	14 (4.8)	191 (4.5)	80 (3.9)		
Residence						
Village	680 (21.6)	44 (15.2)	1033 (24.3)	470 (23.1)	22.679	0.007
Upazila town	432 (13.7)	41 (14.2)	560 (13.2)	276 (13.6)		
District-level town	731. (23.2)	80 (27.7)	943 (22.2)	499 (24.5)		
Divisional city	1308 (41.5)	124 (42.9)	1720 (40.4)	789 (38.8)		
Marital status						
Single	1980 (62.8)	194 (67.1)	3025 (71.1)	1662 (81.7)	223.616	< 0.001
Married	1121 (35.6)	95 (32.9)	1187 (27.9)	350 (17.2)		
Divorced/widowed	39 (1.2	–	39 (0.9)	18 (0.9)		
Others	11 (0.3)	–	5 (0.1)	4 (0.2)		
Someone coming home from COVID-19 affected country						
Yes	87 (2.8)	6 (2.1)	95 (2.2)	57 (2.8)	3.075	0.380
No	3064 (97.2)	283 (97.9)	4161 (97.8)	1977 (97.2)		
Behavior-related variables						
Smoker						
Yes	650 (20.6)	32 (11.1)	507 (11.9)	260 (12.8)	122.083	< 0.001
No	2501 (79.4)	257 (88.9)	3749 (88.1)	1774 (87.2)		
Alcohol consumer						
Yes	115 (3.6)	7 (2.4)	74 (1.7)	62 (3.0)	27.198	< 0.001
No	3036 (96.4)	282 (97.6)	4182 (98.3)	1972 (97.0)		
Social media user						
Yes	2766 (87.8)	279 (96.5)	3941 (92.6)	1968 (96.8)	149.249	< 0.001
No	385 (12.2)	10 (3.5)	315 (7.4)	66 (3.2)		
Lockdown-related questions						
Self-isolation						
Not a single day	289 (9.2)	23 (8.0)	284 (6.7)	142 (7.0)	19.906	0.003
Less than 4 days	164 (5.2)	16 (5.5)	196 (4.6)	98 (4.8)		
4 days or more	2698 (85.6)	250 (86.5)	3776 (88.7)	1794 (88.2)		
Had face-to-face contact with another person for 15 min or more						
Not a single day	1497 (47.5)	141 (48.8)	2188 (51.4)	1078 (53.0)	35.121	< 0.001
Less than 4 days	1104 (35.0)	110 (38.1)	1494 (35.1)	644 (31.7)		
4 days or more	550 (17.5)	38 (13.1)	574 (13.5)	312 (15.3)		
Been outside for 15 min						
Not a single day	1444 (45.8)	134 (46.4)	2136 (50.2)	1033 (50.8)	44.216	< 0.001
Less than 4 days	1001 (31.8)	112 (38.8)	1387 (32.6)	635 (31.2)		
4 days or more	706 (22.4)	43 (14.9)	733 (17.2)	366 (18.0)		
Have enough food supply						
Agree	593 (18.8)	59 (20.4)	845 (19.9)	437 (21.5)	24.014	< 0.001
Disagree	1313 (41.7)	110 (38.1)	1567 (36.8)	745 (36.6)		
Undecided	1245 (39.5)	120 (41.5)	1844 (43.3)	852 (41.9)		
May face economic hardship						
Agree	1441 (56.5)	132 (55.7)	1772 (53.4)	852 (54.8)	8.290	0.218
Disagree	451 (17.7)	40 (16.9)	587 (17.7)	261 (16.8)		
Undecided	660 (25.9)	65 (27.4)	960 (28.9)	441 (28.4)		
Panic of probable economic recession						
Agree	2716 (86.2)	250 (86.5)	3787 (89.0)	1816 (89.3)	25.057	< 0.001
Disagree	202 (6.4)	22 (7.6)	250 (5.9)	122 (6.0)		
Undecided	233 (7.4)	17 (5.9)	219 (5.1)	96 (4.7)		
Health-related variables						
Depressive symptoms						
Non-depressed	2147 (68.1)	202 (69.9)	2845 (66.8)	1284 (63.1)	15.898	0.001
Depressed	1004 (31.9)	87 (30.1)	1411 (33.2)	750 (36.9)		
Self-rated health						
Good	2177 (69.1)	206 (71.3)	2967 (69.7)	1379 (67.8)	7.820	0.252
Fair	864 (27.4)	71 (24.6)	1173 (27.6)	589 (29.0)		
Poor	110 (3.5)	12 (4.2)	116 (2.7)	66 (3.2)		
Chronic disease						
No	2313 (73.4)	210 (72.7)	3313 (77.8)	1562 (76.8)	22.038	< 0.001
Yes	838 (26.6)	79 (27.3)	943 (22.2)	472 (23.2)		
Suicidal ideation						
Yes	147 (4.7)	17 (5.9)	186 (4.4)	138 (6.8)	18.350	< 0.001
No	3004 (95.3)	272 (94.1)	4070 (95.6)	1896 (93.2)		

### Association between study variables and nightly sleep duration

The associations between nightly sleep duration and the study variables are presented in Table 3. No significant association was observed between gender and nightly sleep duration. Non-smokers (χ^2^ = 43.558,* p* < 0.001) and those self-isolating for four days or more (χ^2^ = 16.503,* p* = 0.002) reported significantly more nightly sleep than their counterparts. In addition, not suffering from depressive symptoms (χ^2^ = 15.606, *p* < 0.001), and not having suicidal ideation (χ^2^ = 12.134, *p* = 0.002) reported significantly higher nightly sleep duration than their counterparts.

**Table 3:** Associations between the study variables and night sleep duration.

Variable	Night Sleep duration			Statistics	
	7–9 h (6296, 64.7%); n (%)	< 7 h (2882, 29.6%); n (%)	> 9 h (552, 5.7%); n (%)	*χ*^*2*^ value	*p*-value
Socio-demographic variables					
Age					
18–25 years	3978 (63.2)	1798 (62.4)	391 (70.8)	22.411	< 0.001
26–35 years	1656 (26.3)	729 (25.3)	121 (21.9)		
More than 35 years	662 (10.5)	355 (12.3)	40 (7.2)		
Gender					
Male	3499 (55.6)	1651 (57.3)	302 (54.7)	2.8111	0.590
Female	2787 (44.3)	1227 (42.6)	249 (45.1)		
Transgender	10 (0.2)	4 (0.1)	1 (0.2)		
Occupation					
Employed	1652 (26.2)	782 (27.1)	113 (20.5)	32.428	< 0.001
Student	3678 (58.4)	1649 (57.2)	347 (62.9)		
Retired	25 (0.4)	22 (0.8)	5 (0.9)		
Housewife	458 (7.3)	203 (7.0)	28 (5.1)		
Unemployed	224 (3.6)	91 (3.2)	33 (6.0)		
Other	259 (4.1)	135 (4.7)	26 (4.7)		
Residence					
Village	1462. (23.2)	653 (22.7)	112 (20.3)	8.549	0.201
Upazila town	841 (13.4)	375 (13.0)	93 (16.8)		
District-level town	1465 (23.3)	670 (23.2)	118 (21.4)		
Divisional city	2528 (40.2)	1184 (41.1)	229 (41.5)		
Marital status					
Single	4394 (69.8)	2059 (71.4)	408 (73.9)	7.042	0.317
Married	1826 (29.0)	788 (27.3)	139 (25.2)		
Divorced/widowed	63 (1.0)	28 (1.0)	5 (0.9)		
Others	13 (0.2)	7 (0.2)	–		
Someone coming home from COVID-19 affected country					
Yes	163 (2.6)	56 (1.9)	26 (4.7)	14.817	0.001
No	6133 (97.4)	2826 (98.1)	526 (95.3)		
Behavior -related variables					
Smoker					
Yes	1002 (15.9)	334 (11.6)	113 (20.5)	43.558	< 0.001
No	5294 (84.1)	2548 (88.4)	439 (79.5)		
Alcohol consumer					
Yes	168 (2.7)	69 (2.4)	21 (3.8)	3.588	0.166
No	6128 (97.3)	2813 (97.6)	531 (96.2)		
Social media user					
Yes	5781 (91.8)	2654 (92.1)	519 (94.0)	3.374	0.185
No	515 (8.2)	228 (7.9)	33 (6.0)		
Lockdown-related questions					
Self-isolation					
Not a single day	499 (7.9)	212 (7.4)	27 (4.9)	16.503	0.002
Less than 4 days	292 (4.6)	165 (5.7)	17 (3.1)		
4 days or more	5505 (87.4)	2505 (86.9)	508 (92.0)		
Had face-to-face contact with another person for 15 min or more					
Not a single day	3231 (51.3)	1393, 48.3	280, 50.7	11.628	0.020
Less than 4 days	2098 (33.3)	1065 (37.0)	189 (34.2)		
4 days or more	967 (15.4)	424 (14.7)	83 (15.0)		
Been outside for 15 min					
Not a single day	3100 (49.2)	1368 (47.5)	279 (50.5)	3.229	0.520
Less than 4 days	2013 (32.0)	950 (33.0)	172 (31.2)		
4 days or more	1183 (18.80)	564 (19.6)	101 (18.3)		
Have enough food supply					
Agree	1295 (20.6)	535 (18.6)	104 (18.8)	8.303	0.081
Disagree	2428 (38.6)	1105 (38.30	202 (36.6)		
Undecided	2573 (40.9)	1242 (43.1)	246 (44.6)		
May face economic hardship					
Agree	2738 (55.1)	1212 (53.4)	247 (57.7)	13.687	0.008
Disagree	828 (16.7)	449 (19.8)	62 (14.5)		
Undecided	1400 (28.2)	607 (26.8)	119 (27.8)		
Panic of probable economic recession					
Agree	5574 (88.5)	2529 (87.8)	466 (84.4)	9.529	0.049
Disagree	367 (5.8)	181 (6.3)	48 (8.7)		
Undecided	355 (5.6)	172 (6.0)	38 (6.9)		
Health-related variables					
Depressive symptoms					
Non-depressed	4223 (67.1)	1930 (67.0)	325 (58.9)	15.606	< 0.001
Depressed	2073 (32.9)	952 (33.0)	227 (41.1)		
Self-rated health					
Good	4322 (68.6)	2045 (71.0)	362 (65.6)	13.721	0.008
Fair	1779 (28.3)	756 (26.2)	162 (29.3)		
Poor	195 (3.1)	81 (2.8)	28 (5.1)		
Chronic disease					
No	4795 (76.2)	2195 (76.2)	408 (73.9)	1.443	0.486
Yes	1501 (23.8)	687 (23.8)	144 (26.1)		
Suicidal ideation					
Yes	306 (5.0)	137 (4.8)	45 (8.2)	12.134	0.002
No	5990 (95.0)	2745 (95.2)	507 (91.8)		

7–9 h, recommended sleep duration; < 7 h, shorter sleep duration than recommended; > 9 h, longer sleep duration than recommended.

### Linear regression between selected variables and total daily sleep duration

Table 4 presents the linear regression between selected variables and total daily sleep duration. Results showed that participants who were aged 18–25 years (coefficient = 19.97, *p* < 0.001), aged 26–35 years (coefficient = 10.45, *p* = 0.014), unemployed (coefficient = 20.66, *p* = 0.004), married (coefficient = 11.22, *p* < 0.001), self-isolated 4 days or more (coefficient = 9.08, *p* = 0.028), had face-to-face contact with another person for 15 min or more for four days or more weekly (coefficient = 7.79, *p* = 0.024), assumed they would face economic hardship (coefficient = 7.51, *p* = 0.008), and had depressive symptoms (coefficient = 4.76, *p* = 0.031) were significant predictors of total daily sleep time. The model explained a 2.46% variance to predict total daily sleep duration (*p* < 0.001).

**Table 4:** Linear regression between study variables and total daily sleep duration.

Variable	R^2^ = 0.0246, adj R^2^ = 0.0219, F (_21, 7640_) = 9.18, *p* < 0.001			
	Coefficient	SE	95% CI	*p-*value
Constant	414.27	26.45	362.41 to 466.14	< 0.001
Age				
18–25 years	19.97	5.22	9.71 to 30.22	< 0.001
26–35 years	10.45	4.24	2.14 to 18.76	0.014
More than 35 years	Ref.			
Gender				
Male	26.27	25.30	− 23.32 to 75.87	0.299
Female	31.12	25.31	− 18.50 to 80.75	0.219
Transgender	Ref.			
Occupation				
Employed	− 8.20	4.87	− 17.75 to 1.34	0.092
Student	8.28	5.07	− 1.65 to 18.22	0.102
Retired	16.60	14.27	− 11.37 to 44.59	0.245
Housewife	− 8.29	6.83	− 21.70 to 5.10	0.225
Unemployed	20.66	7.19	6.55 to 34.77	0.004
Other	Ref.			
Marital status				
Single	Ref.			
Married	11.22	3.17	5.01 to 17.44	< 0.001
Divorced/widowed	− 2.44	11.22	− 24.45 to 19.56	0.828
Others	− 36.79	21.49	− 78.91 to 5.33	0.087
Social media user				
Yes	5.34	4.66	− 3.80 to 14.49	0.252
No	Ref.			
Self-isolation				
Not a single day	Ref.			
Less than 4 days	− 0.60	5.66	− 11.71 to 10.50	0.915
4 days or more	9.08	4.13	0.96 to 17.17	0.028
Had face-to-face contact with another person for 15 min or more				
Not a single day	Ref.			
Less than 4 days	− 1.09	2.43	− 5.87 to 3.67	0.652
4 days or more	7.79	3.46	1.00 to 14.58	0.024
May face economic hardship				
Agree	7.51	2.82	1.97 to 13.06	0.008
Undecided	6.42	3.17	0.20 to 12.65	0.043
Disagree	Ref.			
Depressive symptoms				
Non-depressed	Ref.			
Depressed	4.76	2.21	0.42 to 9.10	0.031
Suicidal ideation				
Yes	6.89	4.75	− 2.42 to 16.21	0.147
No	Ref.			

SE, standard error; CI, confidence interval.

### Multinomial logistic regression between selected variables and nap duration

Table 5 presents a multinominal logistic regression between selected variables and naptime. Participants aged 18–25 years, and 26–35 years were 1.87 (CI: 1.31–2.66) and 1.45 (CI: 1.07–1.97) times higher risk of taking more than an hour’s nap daily compared to those aged more than 35 years. Retired individuals were more likely to take more than an hour's nap daily than other professionals (RRR: 3.72, CI: 1.51–9.14). In addition, smokers were less likely to take naps compared to those who were not (RRR: 0.55, CI: 0.36–0.85 for less than 30 min; RRR: 0.65, CI: 0.56–0.75 for 30 to 60 min; RRR: 0.65, CI: 0.54–0.78 for more than 60 min). Social media users were 5.71 times (CI: 2.76–11.81), 1.55 times (CI: 1.26–1.90), and 2.15 times (CI: 1.56–2.98) higher risk of taking a nap less than 30 min daily, 30–60 min daily, and more than an hour daily respectively compared to non-users. Moreover, participants who did not think they would have enough food to get through the pandemic were at higher risk of taking a nap 30–60 min daily than those who did (RRR: 1.12, CI: 1.01–1.25).

**Table 5:** Multinomial logistic regression between the study variables and naptime.

Variable	Naptime					
	Less than 30 min		30–60 min		More than 60 min	
	RRR (95% CI)	*p-*value	RRR (95% CI)	*p-*value	RRR (95% CI)	*p-*value
Socio-demographic variables						
Age						
18–25 years	0.44 (0.23–0.83)	0.012	0.92 (0.72–1.18)	0.541	1.87 (1.31–2.66)	< 0.001
26–35 years	0.74 (0.46–1.21)	0.241	0.95 (0.78–1.15)	0.609	1.45 (1.07–1.97)	0.017
More than 35 years	Ref.					
Gender						
Male	0.19 (0.037–1.06)	0.059	1.94 (0.54–6.92)	0.304	5.70 (0.66–49.27)	0.087
Female	0.29 (0.05–1.59)	0.157	2.30 (0.64–8.21)	0.198	6.56 (0.75–56.73)	0.113
Transgender	Ref.					
Occupation						
Employed	0.61 (0.33–1.14)	0.124	0.65 (0.51–0.83)	0.001	0.60 (0.44–0.81)	0.001
Student	1.47 (0.77–2.79)	0.238	1.15 (0.90–1.49)	0.253	1.29 (0.95–1.77)	0.100
Retired	2.73 (0.51–14.51)	0.239	2.20 (0.82–1.50)	0.038	3.72 (1.51–9.14)	0.004
Housewife	1.36 (0.65–2.86)	0.404	1.10 (0.82–1.50)	0.500	0.66 (0.43–1.02)	0.062
Unemployed	0.89 (0.35–2.25)	0.821	1.05 (0.75–1.48)	0.755	1.42 (0.95–2.13)	0.082
Others	Ref.					
Marital status						
Single	Ref.					
Married	0.95 (0.63–1.40)	0.781	0.95 (0.81–1.10)	0.522	0.85 (0.70–1.04)	0.129
Divorced/widowed	1.93e−07 (–)	0.990	0.92 (0.56–1.50)	0.750	1.63 (0.87–3.05)	0.122
Other	1.98e−07 (–)	0.993	0.43 (0.14–1.29)	0.135	0.62 (0.18–2.06)	0.439
Behavior-related variables						
Smoker						
Yes	0.55 (0.36–0.85)	0.008	0.65 (0.56–0.75)	< 0.001	0.65 (0.54–0.78)	< 0.001
No	Ref.					
Alcohol consumer						
Yes	1.14 (0.49–2.65)	0.747	0.73 (0.53–1.00)	0.056	1.22 (0.86–1.74)	0.252
No	Ref.					
Social media user						
Yes	5.71 (2.76–11.81)	< 0.001	1.55 (1.26–1.90)	< 0.001	2.15 (1.56–2.98)	< 0.001
No	Ref.					
Lockdown-related questions						
Had face-to-face contact with another person for 15 min or more						
Not a single day	Ref.					
Less than 4 days	0.96 (0.60–1.54)	0.892	1.05 (0.87–1.27)	0.559	0.82 (0.65–1.03)	0.092
4 days or more	1.32 (0.68–2.54)	0.407	1.05 (0.82–1.34)	0.672	1.09 (0.81–1.48)	0.543
Been outside for 15 min						
Not a single day	Ref.					
Less than 4 days	1.61 (1.00–2.59)	0.048	1.11 (0.91–1.35)	0.278	1.28 (1.01–1.61)	0.036
4 days or more	0.90 (0.47–1.73)	0.767	0.98 (0.77–1.24)	0.904	1.09 (0.81–1.47)	0.538
Have enough food supply						
Agree	1.07 (0.76–1.50)	0.669	1.14 (1.00–1.30)	0.050	1.17 (1.00–1.37)	0.046
Undecided	1.00 (0.75–1.32)	0.994	1.12 (1.01–1.25)	0.027	1.02 (0.89–1.17)	0.700
Disagree	Ref.					
Panic of probable economic recession						
Agree	0.80 (0.50–1.29)	0.355	1.04 (0.85–1.27)	0.689	0.98 (0.77–1.25)	0.914
Undecided	0.72 (0.37–1.42)	0.374	0.72 (0.55–0.94)	0.019	0.67 (0.48–0.94)	0.023
Disagree	Ref.					
Health-related variables						
Depressive symptoms						
Non-depressed	Ref.					
Depressed	0.84 (0.63–1.10)	0.219	1.01 (0.91–1.12)	0.797	1.06 (0.93–1.32)	0.334
Chronic disease						
Yes	1.15 (0.86–1.54)	0.342	0.89 (0.79–1.00)	0.068	1.15 (0.99–1.32)	0.051
No	Ref.					
Suicidal ideation						
Yes	1.23 (0.72–2.10)	0.446	0.87 (0.69–1.10)	0.274	1.20 (0.93–1.54)	0.151
No	Ref.					

RRR, relative risk ratio; CI, confidence interval; No nap considered as base outcome.

### Multinomial logistic regression between selected variables and nightly sleep duration

Table 6 presents a multinominal regression between selected variables and nightly sleep duration. It showed that those aged 26–35 years were at lower risk of sleeping less than 7 h nightly than those aged over 35 years (RRR: 0.80, CI: 0.67–0.94) and that those aged 18–25 years were at higher risk of sleeping more than 9 h nightly (RRR: 1.65, CI: 1.06–2.58) compared to aged more than 35 years. Housewives were at a lower risk of sleeping less than 7 h nightly than other occupations (RRR: 0.74, CI: 0.56–0.98). Additionally, cigarette smokers were more likely to sleep more than 9 h nightly compared to non-smokers (RRR: 1.44, CI: 1.15–2.48). Participants self-isolating for four days or more were more likely to sleep more than 9 h nightly compared to those who were not isolated on any day (RRR: 1.66, CI: 1.11–2.48). Furthermore, individuals with depressive symptoms were at 1.28 times higher risk of sleeping more than 9 h nightly than those with no depressive symptoms. Those experiencing suicidal ideation also had increased duration of sleep nightly than those not experiencing suicidal ideation (RRR: 1.46, CI: 1.05–2.05).

**Table 6:** Multinomial logistic regression between study variables and night sleep duration.

Variable	Night sleep duration			
	< 7 h		> 9 h	
	RRR (95% CI)	*p-*value	RRR (95% CI)	*p-*value
Socio-demographic variables				
Age				
18–25 years	0.82 (0.66–1.000)	0.055	1.65 (1.06–2.58)	0.025
26–35 years	0.80 (0.67–0.94)	0.009	1.17 (0.79–1.75)	0.422
More than 35 years	Ref.			
Occupation				
Employed	0.91 (0.73–1.15)	0.470	0.75 (0.47–1.18)	0.218
Student	0.86 (0.68–1.08)	0.209	0.74 (0.47–1.17)	0.206
Retired	1.44 (0.77–2.69)	0.252	2.46 (0.83–7.33)	0.104
Housewife	0.74 (0.56–0.98)	0.040	0.76 (0.42–1.30)	0.365
Unemployed	0.82 (0.59–1.14)	0.251	1.31 (0.75–2.26)	0.334
Other	Ref.			
Someone coming home from COVID-19 affected country				
No	Ref.			
Yes	0.75 (0.55–1.02)	0.068	1.86 (1.21–2.85)	0.004
Behavior-related variables				
Smoker				
Yes	0.66 (0.58–0.76)	< 0.001	1.44 (1.15–2.48)	0.001
No	Ref.			
Lockdown-related questions				
Self-isolation				
Not a single day	Ref.			
Less than 4 days	1.32 (1.03–1.70)	0.028	1.06 (0.56–1.99)	0.840
4 days or more	1.05 (0.89–1.25)	0.512	1.66 (1.11–2.48)	0.013
Health-related variables				
Depressive symptoms				
Non-depressed	Ref.			
Depressed	1.02 (0.93–1.13)	0.565	1.28 (1.06–1.53)	0.008
Suicidal ideation				
Yes	0.99 (0.80–1.22)	0.930	1.46 (1.05–2.05)	0.024
No	Ref.			

RRR. relative risk ratio; CI, confidence interval; Recommended sleep duration (7–9 h) considered as base outcome.

### Sleep duration during the COVID-19 pandemic by regional divisions

**Figure 1:**
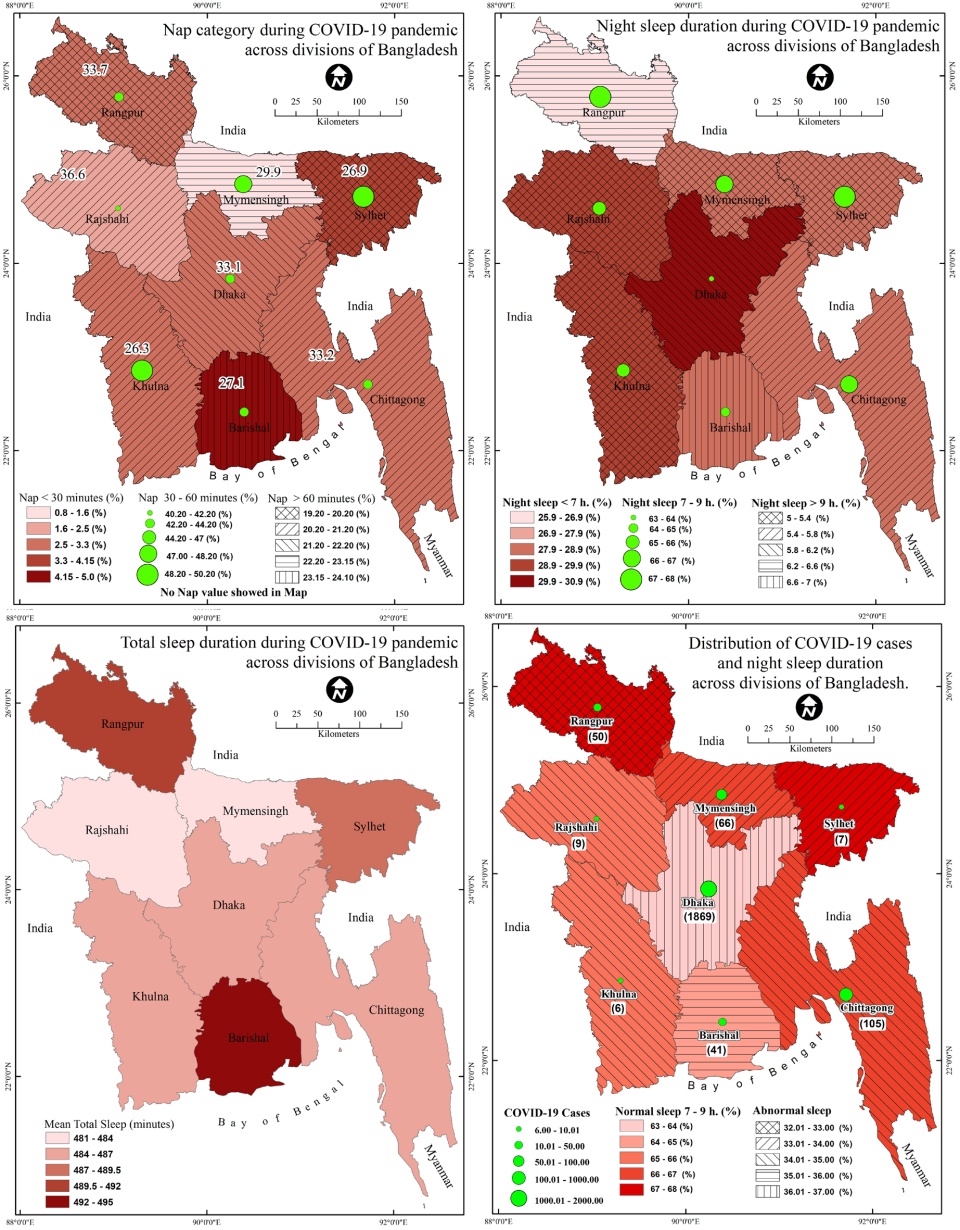
Sleep duration across divisions during the COVID-19 pandemic in Bangladesh.

Results of the regional division-wise sleep duration during the COVID-19 pandemic are presented in Fig. 1. More specifically, nightly sleep duration, daily nap duration, and total daily sleep are displayed across regional divisions. Nap duration was significantly associated with respective regional divisions (χ^2^ = 51.438,* p* < 0.001), whereas night sleep duration, and total sleep duration were not significant (*p* = 0.55 and *p* = 0.58 respectively). Spatial distribution suggested that 24% of participants from the Barisal had a nap duration of more than 1 h followed by Mymensingh (22.30%) and Dhaka (21.40%). A nap duration between 30 and 60 min daily was highest in Sylhet (50.20%), followed by Khulna (49.80%) and Mymensingh (47%). For nightly sleep duration, 67.60% of participants from Rangpur reported 7–9 h, whereas less than 7 h was the highest among the residents of Dhaka. About 7% of residents from Barisal had a night sleep duration of more than 9 h. The mean score for total sleep duration in 24 h was highest among residents from Barisal, while the lowest was found among residents in Rajshahi. It was also found that the participants from regional divisions with higher COVID-19 cases (for example, Dhaka) were more likely to have abnormal sleep status.

## Discussion

The implementation of public health prevention measures such as spatial distancing, self-isolating, and quarantining during the COVID-19 pandemic were introduced to minimize virus transmission until vaccines were introduced. However, such restrictive measures may also increase mental health problems among individuals ^3^. Studies throughout the world have reported higher levels of mental health problems and sleep problems during the pandemic^16,37^. Although a few studies examining COVID-19-related sleep problems have been conducted previously in Bangladesh, none of them established the total sleep duration, night-time sleep, and daily naptime and its associated factors among the Bangladeshi residents during the COVID-19 pandemic alongside GIS-based distributions.

The present study found that 64.7% of participants slept 7–9 h nightly, while 29.6% slept less than 7 h nightly and 5.7% slept more than 9 h nightly. Before the pandemic, a study from Bangladesh^30^ comprising 3968 participants from rural and urban areas observed total daily sleep time. They reported that 87.4% of adults (aged 18–64 years) slept between 7 and 9 h nightly, whereas 8.9% slept less than 7 h nightly and 3.7% slept more than 9 h nightly. In the same study, 41.9%, 55.6%, and 2.5% of school-aged children (aged 6–13 years) slept between 9 and 11 h, less than 9 h, and more than 11 h, respectively. Using 8–10 h as recommended nightly sleep time, the prevalence rate was 76.1% for teenagers and 56.4% among older adults with a recommended 7–8 h of nightly sleep time^30^. A previous multi-country study^18^ demonstrated a higher prevalence of sleeping less than 7 h nightly during the pandemic (i.e., 35%), whereas the same study noted the rate was 42% before the pandemic. They also reported that more than one-third of the participants reported worsening sleep during the pandemic^18^. Another study conducted among 782 COVID-19 affected community participants in India reported increased sleep duration (before infection: 7.84 ± 1.33 h; after infection: 8.15 ± 2.00 h)^38^. Shorter sleep duration is significantly associated with obesity and diabetes^39^, increased BMI^40^, and telomere damage^12^, whereas longer sleep duration can lead to a better health-related quality of life^41^.

Daily nap duration was reported to be 30–60 min among 43.7% of participants, 20.9% more than 60 min, 3.0% less than 30 min, and 2.40% had no nap at all. A slightly higher nap duration was reported from a neighboring country (India) which reported that 25% of participants napped more than 60 min daily. The study also found a significant differences before and during the COVID-19 pandemic in terms of reporting changes in sleep schedule (31.1% vs. 38% for < 60 min, 9.2% vs. 25% for > 60 min, 59.7% vs. 37% for no naps; all *p-*values < 0.001)^19^. Another study among COVID-19 infected community participants reported that 11.1% took naps less than 30 min, 12.4% took naps of 30–45 min, 10.4% took naps lasting more than 45 min, 14.8% took naps once in a while, and 20.3% took naps during holidays or weekends before getting infected with COVID-19^38^. However, changes in nap pattern were observed after being infected with COVID-19 because 20.2% had longer nap duration compared to their previous nap time^38^.

The present study identified several factors associated with sleep duration such as participants being aged 18–25 years, being unemployed, being married, being self-isolated for four days or more week, being a cigarette smoker, being a social media user, facing economic hardship, having depressive symptoms, and experiencing suicidal ideation. The sleep patterns of young adults changed during the COVID-19 pandemic. The youngest age group (18–24 years old) had the shortest sleep duration before lockdown but this group reported delayed wake-up times and longer sleep duration during the lockdown period compared with other age groups^42^. Similar findings have also been observed in a different study in Bangladesh before the pandemic^30^. Additionally, being married and unemployed was associated with increased sleep time. The previous Bangladeshi study also reported that total sleep time had a significant relationship with marital status and unemployment^30^.

During the COVID-19 pandemic, a significant change has been observed in terms of sleep problems^19,43^. Suffering from mental health problems such as depression has had a strong association with sleep difficulties^19,43^, which is consistent with the present study’s results. However, a recent meta-analysis by Zhai et al.^44^ found that both short sleep duration and long sleep duration were significantly associated with increased risk of depression among adults. The study reported that the pooled relative risk for depression was 1.31 (95% CI: 1.04–1.64), and 1.42 (95% CI: 1.04–1.92) in terms of short and long sleep duration, respectively^44^. The present study reported that participants having depressive symptoms were more likely to have a longer sleep duration (more than 9 h nightly). Similarly, findings from meta-analysis supported that longer sleep duration had a significant association with increased risk of depression^44^. Conversely, the present study’s results differed from other studies reporting short sleep duration as an independent risk factor for depression^45,46^. Therefore, to identify the actual relationship between sleep duration and depressive symptoms, further investigation is required.

In addition, using social media more frequently could potentially affect the sleep cycle. A previous study demonstrated that using social media late at night and high emotional attachment to smartphones was associated with poor sleep quality^47^. Blue light emission produced by smartphones possibly decreases melatonin production and affects circadian rhythms adversely^48^. Additionally, the relationship between social media use and cognitive function depletion during the day was also reported in another study and associated with higher daytime naps^25^.

With respect to GIS-based spatial distribution, nap duration was significantly associated with the respective regional divisions. Findings suggested that participants from Barisal had a nap duration of more than 1 h, whereas, 30–60 min nap duration was highest among the participants from Sylhet. For nightly sleep duration, those in Rangpur and Sylhet had higher normal sleep status (7–9 h), whereas Dhaka had the lowest prevalence of normal sleep status. The number of COVID-19 cases in Dhaka, the capital of the country, was the highest at the time of the survey. It is likely that some individuals panicked and feared being infected with COVID-19 resulting in a change in their normal sleep status. The fear of COVID-19 increases among those who are in high-exposure professions such as healthcare professionals^49^, and this cohort has been reported as being one of the most vulnerable to mental health suffering as reported in a recent meta-analysis^50^. Other studies have reported that COVID-19 exposure increases psychological instabilities, whereas fear of COVID-19 partially mediates the association between COVID-19 exposure and depression. Therefore, it is not surprising that abnormal sleep status was associated with high COVID-19 exposure regions compared to other regions with lower COVID-19 exposure (for example, Rangpur, Sylhet).

In addition to the level of COVID-19 exposure, the findings may be explained by the number of people living in a city. For instance, a previous Bangladeshi study found that individuals living in a large city (e.g., Dhaka) had less sleep^30^. Dhaka, the largest city in Bangladesh (and one of the most densely populated and unplanned cities worldwide), is in a poor situation with respect to environmental indicators leading to the alteration of normal sleep time. For instance, air pollution concentration has been negatively associated with sleep duration. More specifically, one standard deviation increase in air pollution concentration was reported to be associated with a reduction in total daily hours of sleep by 0.68^51^. As aforementioned, Dhaka is one of the most populated cities worldwide and is the third most air-polluted city worldwide^52^, and it is not unusual to have altered sleep status among this population. The environmental conditions for the cities of Rangpur, Sylhet and Chittagong, are better with less exposure to air pollution which may also be another explanation for the higher rates of normal sleep status in these cities.

## Implications of the findings

The present study included a large sample size as well as individuals from every regional division in the country, both of which may potentially increase the generalizability of the findings. For the first time, a regional distribution was provided in terms of sleep duration during the pandemic that may be used to identify in which regions people are more susceptible to abnormal sleep duration. The geolocated findings can therefore facilitate longitudinal studies by enabling recruitment of susceptible individuals from a particular geographical area to assess the long-term effects of abnormal sleep on their quality of life.

## Limitations

The present study has some limitations. The study was cross-sectional. Therefore, it cannot provide any determination of causality between the variables. The study was carried out utilizing online platforms due to the restrictions due to the COVID-19 pandemic, which limited face-to-face interaction. Consequently, more than half of the participants belonged to the student cohort due to their availability online, indicating that the sample was not nationally representative. Moreover, the present study did not consider other important predictors of sleep disturbances, such as the (1) history of sleep problems before the COVID-19 pandemic, (2) use of specific sleep medications, (3) late-night activities and habits, and (4) engagement with smartphones. There may also be a selection bias because the data were collected using an online platform, and the sample was self-selecting.

## Conclusions

This study is the first to provide sleep time mapping in Bangladesh. The present study concluded that during the COVID-19 pandemic more people had abnormal sleep duration compared to before the pandemic in Bangladesh (i.e., 29.6% vs 8.9% slept less than 7 h nightly; and 5.7% vs 3.7% slept more than 9 h nightly). In addition, a number of associated factors of abnormal sleep status were identified (e.g., age, unemployment, marital status, cigarette smoking, depression, suicidal ideation etc.), and individuals living in areas of high COVID-19 exposure areas reported higher levels of sleep abnormality. These findings are helpful for the respective regional divisional authorities undertaking any mental health interventions aimed at prevention and management strategies to support vulnerable regions and sectors of the population.

## Supplementary Information

Supplementary Information.

## Data Availability

Data are available from the corresponding author upon reasonable request.
