# Supplementary material for: Sleep duration during the COVID-19 pandemic in Bangladesh: A GIS-based large sample survey study
Source: Sci Rep. 2023 Feb 27;13:3368. doi: 10.1038/s41598-023-30023-1 (PMC9969935; doi:10.1038/s41598-023-30023-1)
Supplement: Supplementary file 1 — Supplementary Information. [file 41598_2023_30023_MOESM1_ESM.pdf]

Sleep duration during the COVID-19 pandemic in Bangladesh: A GIS-based large sample survey study

Firoj Al-Mamun[1,2,3*], Nur Hussain[1], Najmuj Sakib[4], Ismail Hosen[1,2], Istihak Rayhan[5], Abu Hasnat Abdullah[1,2], AKM Israfil Bhuiyan[1], Md. Abedin Sarker[1,2], Sahadat Hossain[2,6], Liye Zou[7], Md. Dilshad Manzar[8], Chung-Ying Lin[9], Md. Tajuddin Sikder[2], Mohammad Muhit[3], Amir H. Pakpour[10], David Gozal[11], Mark D. Griffiths[12], and Mohammed A. Mamun[1,2,3,13*]

1. CHINTA Research Bangladesh, Savar, Dhaka, Bangladesh.
2. Department of Public Health and Informatics, Jahangirnagar University, Savar, Dhaka, Bangladesh
3. Department of Public Health, University of South Asia, Dhaka, Bangladesh
4. Department of Microbiology, Jashore University of Science and Technology, Jashore, Bangladesh
5. Department of Economics, Jahangirnagar University, Savar, Dhaka, Bangladesh
6. Department of Behavioural Science and Health, Institute of Epidemiology and Health Care, University College London (UCL), London, England
7. Body-Brain-Mind Laboratory, School of Psychology, Shenzhen University, Shenzhen, China
8. Department of Nursing, College of Applied Medical Sciences, Majmaah University
9. Institute of Allied Health Sciences, College of Medicine, National Cheng Kung University, Tainan, Taiwan
10. Social Determinants of Health Research Center, Research Institute for Prevention of Non-Communicable Diseases, Qazvin University of Medical Sciences, Qazvin, Iran
11. Department of Child Health and the Child Health Research Institute, The University of Missouri School of Medicine, Columbia, MO, United States
12. International Gaming Research Unit, Psychology Department, Nottingham Trent University, Nottingham, United Kingdom
13. Department of Public Health, Daffodil International University, Dhaka, Bangladesh

*** Correspondence**
Firoj Al-Mamun, CHINTA Research Bangladesh, Dhaka, Bangladesh. Email: firojphiju@gmail.com, and

Mohammed A. Mamun, CHINTA Research Bangladesh, Dhaka, Bangladesh. Email: mamunphi46@gmail.com

Do you want to participate in this survey?

1. Yes
2. No

Socio-demographics (SD)

SD_1: Your age (Write in number, e.g. 20)

…………………………..

SD_2: Gender

1. Male
2. Female
3. Others

SD_3: Occupation

1. Unemployed
2. Day-laborer
3. Farmer
4. Business
5. Student
6. Government employee
7. Private employee
8. Retired
9. Housewife
10. Others

SD_4: Current place of residence (e.g. Dhaka district)

…………………………..

SD_5: Which type of administrative region are you living in?

1. Village
2. Upazilla town
3. District-level town
4. Divisional city

SD_6: Marital status

1. Unmarried
2. Married
3. Divorced/Widower/Widow
4. Others

SD_7: Did anyone come to your home from any COVID-19 affected countries after January 2020?

1. Yes
2. No

Behavioral health-related information (BH)

BH_1: Do you smoke?

1. Yes
2. No

BH_2: Do you consume alcohol?

1. Yes
2. No

BH_3: Do you use social media like Facebook, Twitter, WhatsApp or others?
1. Yes
2. No

BH_4: What do you think of your current health condition?
1. Good
2. Fair
3. Poor

BH_5: Are you suffering from any chronic disease? (You can choose more than one answer) (If you do not have any, please proceed to the next question)
1. Diabetics
2. High blood pressure
3. Asthma/ Respiratory problem
4. Heart disease
5. Kidney problem
6. Cancer
7. Any other not listed
8. Not suffering from any chronic disease

BH_6: What time do you generally go to sleep at night? (e.g. 11:00 pm)
……………………..

BH_7: What time do you wake up usually? (e.g. 6:30 am)
…………………………

BH_8: Do you take naps during the day?
1. Yes
2. No

BH_9: If "YES", how long do you take naps? (Write in minutes, e.g. 30) (If your previous answer is 'NO', skip the question)
………………………………….

Lockdown-related questions (LRQ)

LRQ_1: How many days were you self-isolated?
1. Not a single day
2. Less than 4 days
3. 4 days or more

LRQ_2: How many days you had face-to-face contact with another person for 15 minutes or more?
1. Not a single day
2. Less than 4 days
3. 4 days or more

LRQ_3: How many days you had been outside for15 minutes?
1. Not a single day
2. Less than 4 days

3. 4 days or more

LRQ_4: If this lockdown situation persists more than a month, do you think you will have enough food supply?

1. Agree
2. Disagree
3. Undecided

LRQ_5: Are you panicked of any probable economic recession due to this pandemic?

1. Agree
2. Disagree
3. Undecided

LRQ_6: If you are a wage earner, do you think you may lose your job and/or face economic hardship?

1. Agree
2. Disagree
3. Undecided

Patient health Questionnaire (PHQ-9) scale

Answer to the following statements regarding your current status (i.e. last 2 weeks) using a four-item Likert-type scale.
Answers included: 1. Not at all 2. Several days 3. More than half the days 4. Nearly every day

PHQ-9_1: Little interest or pleasure in doing things
PHQ-9_2: Feeling down, depressed or hopeless
PHQ-9_3: Trouble falling or staying asleep, or sleeping too much
PHQ-9_4: Feeling tired or having little energy
PHQ-9_5: Poor appetite or overheating
PHQ-9_6: Feeling bad about yourself-or that you are a failure or have let yourself or your family down
PHQ-9_7: Trouble concentrating on things, such as reading the newspaper or watching television
PHQ-9_8: Moving or speaking so slowly that other people could have noticed. Or the opposite-being so fidgety or restless that you have been moving around a lot more than usual
PHQ-9_9: Thoughts that you would be better off dead, or of hurting yourself

Suicide related questions (SRQ)

SRQ: Do you think about committing suicide, and are these thoughts persistent and related to COVID-19 issues?

1. **Yes**
2. **No**
